# Longitudinal serum albumin trajectories and neurological outcomes after aneurysmal subarachnoid hemorrhage

**DOI:** 10.3389/fnut.2026.1979070

**Published:** 2026-10-19

**Authors:** Hua Tang, Yanli Tang, Siqi Wu, Qunxiang Zhang, Youwen Gong

**Affiliations:** Department of Neurosurgery, Changde Hospital, Xiangya School of Medicine, Central South University (The First People’s Hospital of Changde City), Changde, Hunan, China

**Keywords:** aneurysmal subarachnoid hemorrhage, latent class mixed model, neurological outcome, nutrition, serum albumin, trajectory

## Abstract

**Background:**

Serum albumin is routinely measured after aneurysmal subarachnoid hemorrhage (aSAH), but most prognostic studies have relied on single measurements. We investigated whether longitudinal albumin patterns during the acute phase were associated with neurological outcome.

**Methods:**

This retrospective cohort study screened 490 patients with intracranial aneurysms and 498 admissions. After restricting to the index hospitalization for strictly defined aSAH, 370 patients remained. The trajectory cohort included 318 patients with serum albumin measured within 48 h of admission and on at least three different days during days 0–14. Latent class mixed models identified albumin trajectories. The primary outcome was unfavorable neurological status at discharge (modified Rankin Scale [mRS] ≥ 3). Multivariable logistic regression, restricted cubic spline analyses, landmark analyses using early albumin windows, and multiple sensitivity analyses addressing severity, treatment modality, missing data, hospitalization duration, and latent-class uncertainty were performed.

**Results:**

Three trajectories were identified: high-preserved (*n* = 90, 28.3%), high-declining (*n* = 148, 46.5%), and low-persistent (*n* = 80, 25.2%). Overall, 114 patients (35.8%) had unfavorable neurological outcomes. In the principal complete-case model (*n* = 258), compared with the high-preserved group, the adjusted odds ratios were 3.65 (95% CI, 1.57–8.47; *p* = 0.003) for the high-declining trajectory and 2.48 (95% CI, 1.02–6.02; *p* = 0.044) for the low-persistent trajectory (global *p* = 0.007). Early-window landmark analyses showed that higher day-3 and day-7 nadir albumin levels were associated with lower odds of subsequent unfavorable discharge status (OR per 1 g/L increase, 0.86 and 0.84, respectively; both *p* < 0.001). Treatment-stratified analyses showed no evidence of trajectory-by-treatment interaction (*p* = 0.453). Accounting for latent-class uncertainty yielded consistent results, although the low-persistent estimate became less precise. Adding trajectory class to the clinical model resulted in only modest improvement in discrimination (C-statistic 0.754 to 0.776).

**Conclusion:**

Longitudinal serum albumin patterns were associated with neurological status at discharge after aSAH. However, because trajectory assignment incorporates post-baseline measurements and class separation was moderate, these trajectories should be interpreted as markers of evolving clinical course rather than validated independent predictors. Early landmark analyses improved temporal interpretation but did not eliminate residual confounding or reverse causation. As standardized 90-day mRS was unavailable, these findings should not be extrapolated to long-term functional prognosis.

## 1 Introduction

Aneurysmal subarachnoid hemorrhage (aSAH) remains a severe cerebrovascular emergency despite major advances in aneurysm occlusion and neurocritical care. Contemporary guidelines emphasize early aneurysm treatment, prevention of rebleeding, surveillance for delayed cerebral ischemia, and coordinated neurocritical care, yet substantial mortality and disability persist (1, 2). Recent national and multicenter cohorts continue to show marked heterogeneity in recovery, with age, neurological grade, hemorrhage burden, delayed ischemic injury, and other evolving physiological disturbances contributing to outcome (3). This heterogeneity suggests that prognostic assessment based only on admission characteristics may not fully capture the biological course that unfolds during the days after hemorrhage (4).

Serum albumin is particularly relevant because it is inexpensive, routinely available, and closely linked to both nutritional reserve and the systemic response to acute illness. Recent aSAH studies have connected nutritional and albumin-related indices with functional outcome, infection, and disease severity (5). A lower hemoglobin-albumin-lymphocyte-platelet score has been associated with unfavorable functional outcome and postoperative pneumonia, while high nutritional risk assessed with the modified NUTRIC score has been related to mortality, intensive care burden, and functional status (6). Large observational data also suggest that hypoalbuminemia and an early fall in albumin are associated with hospital-acquired infection after aSAH (7). Albumin-containing ratios, including the D-dimer/albumin ratio and albumin/fibrinogen ratio, have likewise shown prognostic associations (8).

These observations fit within a broader literature showing that inflammatory and immune-nutritional markers may capture clinically relevant systemic stress after aSAH. Preoperative inflammatory biomarkers, postoperative systemic immune-inflammation indices (9), C-reactive protein/albumin ratio, serum lactate, peroxiredoxin 2, neurofilament light chain, and pentraxin 3 have all been examined as prognostic markers (10). More recently, machine-learning models integrating clinical, inflammatory, and nutritional indicators have achieved useful short-term discrimination, reinforcing the potential value of combining conventional neurological grading with systemic biomarkers (11).

Most albumin studies, however, have treated albumin as a static exposure or used only a small number of predefined time points. This may miss clinically important differences between patients whose albumin remains relatively preserved, those who enter hospital with low concentrations, and those who begin with apparently preserved albumin but subsequently decline. Recent work using trajectory-based approaches for glycemia and other longitudinal data in aSAH demonstrates that temporal patterns may provide information beyond admission measurements. We therefore aimed to identify longitudinal serum albumin trajectories during the first 14 days after admission and to examine their association with unfavorable neurological outcome at discharge (12). We additionally assessed baseline and nadir albumin concentrations using restricted cubic spline analyses. Because trajectory membership and nadir albumin are defined using post-baseline measurements, these analyses were considered associational rather than causal or independently predictive; additional landmark and sensitivity analyses were used to examine temporal robustness.

## 2 Materials and methods

### 2.1 Study design and data source

This retrospective cohort study was based on routinely collected electronic medical records from a single hospital. The source database contained 490 patients with intracranial aneurysms and 498 hospital admissions between 2021 and 2024. Clinical information was retrieved from linked electronic records, including demographic characteristics, diagnoses, laboratory tests, imaging examinations, surgical and endovascular procedures, admission assessments, daily progress notes, and discharge summaries. For patients with more than one hospitalization, only the first admission meeting the study definition of aneurysmal subarachnoid hemorrhage (aSAH) was retained as the index hospitalization. A strict aSAH definition was used for the primary cohort, requiring documentation of a ruptured intracranial aneurysm during the index hospitalization together with the corresponding subarachnoid hemorrhage-related clinical record. After application of this definition, 370 patients were eligible for further screening.

### 2.2 Study population

Patients were included in the longitudinal albumin analysis when they met all of the following criteria: (1) a strict diagnosis of aSAH during the index hospitalization; (2) at least one valid serum albumin measurement within 48 h after admission; and (3) serum albumin measurements available on at least three different calendar days during the first 14 days after admission. Patients without an albumin measurement within the first 48 h or with measurements on fewer than three different days during days 0–14 were excluded from the trajectory analysis. Of the 370 patients with strictly defined aSAH, 4 had no serum albumin measurement within 48 h and 48 had fewer than three measurement days during the 14-day observation period. The final trajectory cohort therefore consisted of 318 patients.

### 2.3 Serum albumin measurements

Serum albumin measurements obtained as part of routine biochemical testing were extracted from the electronic laboratory system. Time was calculated relative to the date of hospital admission, with the admission day defined as day 0. Measurements obtained between day 0 and day 14 were used for trajectory modeling. When more than one albumin measurement was available on the same calendar day, the measurements were averaged to obtain one value for that patient-day. This resulted in one longitudinal observation per patient per measurement day. Patients contributed all available daily albumin values within the 14-day observation window; measurements were not required to occur at identical time points across patients. For supplementary analyses, baseline albumin was defined as the earliest available daily albumin value within 48 h after admission. Nadir albumin was defined as the lowest daily serum albumin concentration recorded between day 0 and day 14.

### 2.4 Identification of longitudinal albumin trajectories

Latent class mixed models were used to identify distinct longitudinal patterns of serum albumin during the first 14 days after admission. All models included a patient-specific random intercept to account for within-patient correlation arising from repeated measurements. Because the shape of albumin change was not assumed to be linear, several alternative specifications for time were examined, including linear, quadratic, cubic, and natural cubic spline functions. Natural cubic spline models with three and four degrees of freedom were also evaluated. For the more flexible time specification, solutions containing two, three, and four latent classes were fitted.

Model selection was based on a combination of statistical fit and classification quality rather than on a single information criterion. The Akaike information criterion (AIC), Bayesian information criterion (BIC), change in BIC relative to the best-fitting solution, entropy, average posterior probability of class membership, class size, and clinical interpretability of the resulting trajectories were considered. A BIC difference of <2 between competing models was treated as weak evidence favoring one model on fit alone. Solutions containing very small classes were considered unstable and were not preferred. Average posterior probabilities above approximately 0.70 were considered acceptable, while entropy was interpreted as a global measure of separation rather than a pass/fail criterion.

After comparison of the candidate models, a three-class model using a natural cubic spline with four degrees of freedom and a random intercept was retained as the selected working solution. Each patient was assigned to the class for which the posterior probability of membership was highest. To characterize classification uncertainty more fully, we additionally summarized class-specific mean and median assigned posterior probabilities, the proportions with assigned posterior probability ≥0.70 and ≥0.80, and odds of correct classification. For Figure 1, class-specific natural cubic spline curves were refitted conditional on assigned class, with patient-clustered robust standard errors used to derive 95% confidence intervals; the number of patients contributing an albumin measurement on each study day was also displayed. The resulting trajectories were labeled high-preserved, high-declining, and low-persistent according to their observed patterns.

**Figure 1:**
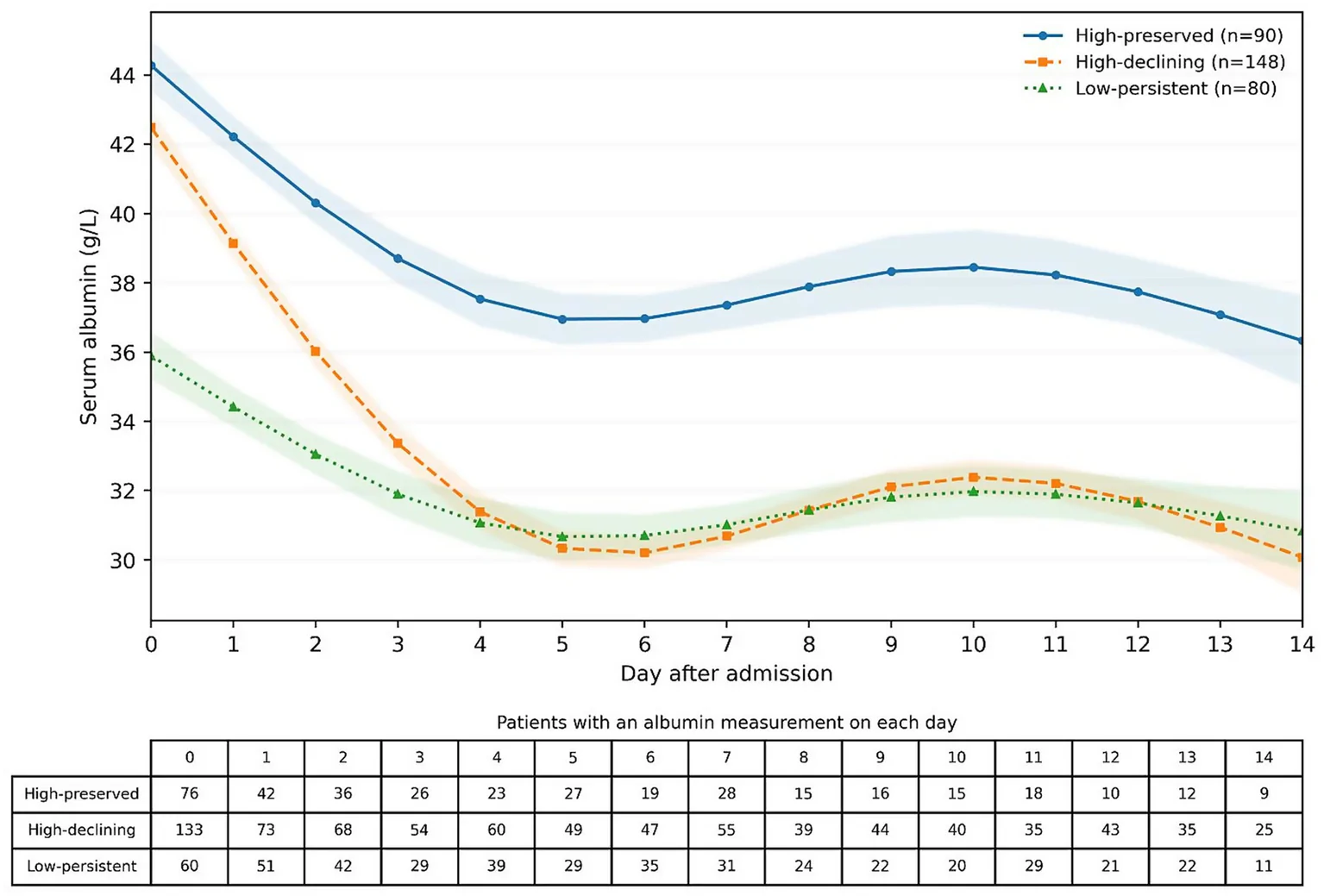
Selected three-class longitudinal serum albumin patterns during the first 14 days after admission. Curves are class-specific natural cubic spline estimates conditional on assigned latent class, and shaded bands show 95% confidence intervals based on patient-clustered robust standard errors. The table reports the number of patients contributing an albumin measurement on each study day. Confidence intervals describe uncertainty in the class-specific curves conditional on assignment and do not incorporate uncertainty in latent-class membership.

### 2.5 Clinical variables and covariates

Demographic and clinical variables were obtained from admission records, diagnostic records, procedural data, and laboratory results. Variables considered in the analysis included age, sex, history of hypertension, admission Glasgow Coma Scale (GCS) score, Hunt–Hess grade, modified Fisher grade, treatment modality, and baseline serum albumin. Treatment was categorized as microsurgical clipping, endovascular treatment, or both treatments during the index hospitalization. A pragmatic baseline severity indicator was used in the principal multivariable analysis. Poor neurological grade was defined primarily as Hunt–Hess grade 3–5; only when Hunt–Hess grade was unavailable was admission GCS ≤ 12 used as a fallback indicator. Hunt–Hess and GCS were not assumed to be interchangeable measures. A sensitivity analysis was therefore restricted to patients with an available Hunt–Hess grade. Covariates for multivariable modeling were selected on the basis of clinical relevance and potential confounding rather than solely according to significance in univariable analysis.

### 2.6 Neurological outcome assessment

The primary outcome was unfavorable neurological status at hospital discharge, defined as a modified Rankin Scale (mRS) score ≥3. An mRS score of 0–2 was considered a favorable outcome. When an mRS score was explicitly documented in the discharge summary or final clinical record, the recorded value was used. For patients without an explicitly documented mRS score, discharge functional status was retrospectively adjudicated from the medical records. Two independent investigators, who were trained in the use of the mRS and blinded to the patients’ serum albumin trajectory classifications, independently reviewed the discharge summary and the last available progress notes and assigned an mRS score according to the standard seven-level scale. Disagreements between the two investigators were resolved through discussion and consensus; when consensus could not be reached, a third senior investigator reviewed the medical record and made the final determination. Because standardized 90-day follow-up mRS was not systematically captured in the retrospective source records, discharge mRS was prespecified as a short-term in-hospital endpoint and was not considered equivalent to 90-day functional outcome.

The adjudication considered the patient’s level of consciousness, residual neurological deficits, limb strength, ambulatory ability, dependence in activities of daily living, cognitive or language impairment, swallowing function, and need for enteral nutritional or airway support at discharge. The standard mRS categories were applied: 0, no symptoms; 1, symptoms without significant disability; 2, slight disability but functionally independent; 3, moderate disability requiring some assistance but able to walk without assistance; 4, moderately severe disability with inability to walk or attend to bodily needs without assistance; 5, severe disability requiring constant nursing care; and 6, death. To reduce potential misclassification arising from retrospective reconstruction of functional status, the investigators applied a prespecified adjudication protocol before reviewing the records. Cases in which the distinction between mRS 2 and 3 remained uncertain were specifically discussed during the consensus review. The historical abstraction dataset retained the final consensus mRS but not both investigators’ pre-consensus ratings; consequently, a weighted inter-rater kappa could not be reconstructed retrospectively.

### 2.7 Statistical analysis

Continuous variables are presented as mean ± standard deviation (SD) or median with interquartile range (IQR), and categorical variables as frequencies and percentages. Differences across serum albumin trajectory groups were assessed using one-way analysis of variance, the Kruskal–Wallis test, chi-square test, or Fisher’s exact test, as appropriate. Longitudinal albumin trajectories during days 0–14 were identified using latent class mixed models with a patient-specific random intercept. Linear, quadratic, cubic, and natural cubic spline functions were evaluated, and two- to four-class solutions were compared according to AIC, BIC, entropy, posterior classification diagnostics, class size, and clinical interpretability. The selected three-class solution used a natural cubic spline with four degrees of freedom.

Binary logistic regression was used to examine associations between assigned trajectory class and unfavorable neurological outcome (mRS ≥ 3), with the high-preserved group as the reference. Sequential models adjusted for age, sex, treatment modality, hypertension, and the pragmatic baseline severity indicator; the complete-case Model 3 was the principal adjusted analysis. Restricted cubic spline analyses were additionally performed for baseline albumin and nadir albumin, adjusting for the same baseline covariates. All tests were two-sided, and *p* < 0.05 was considered statistically significant. Analyses were performed in R software (R Foundation for Statistical Computing, Vienna, Austria; version 4.6.1) using the lcmm, splines, rms, mice, pROC, tidyverse, and ggplot2 packages, supplemented by custom resampling functions.

### 2.8 Sensitivity analyses and model evaluation

To strengthen temporal interpretation, landmark analyses were performed using predefined day-3 and day-7 windows. Patients who were discharged or died on or before the landmark were excluded, and at least two albumin measurements within the corresponding window were required. Early nadir albumin from admission through the landmark was used as the exposure, and subsequent discharge mRS ≥ 3 was modeled after adjustment for age, sex, hypertension, treatment modality, and the baseline severity indicator. A separate sensitivity model was restricted to patients with an available Hunt–Hess grade, defining poor grade only as Hunt–Hess 3–5.

Missing baseline severity was further addressed by multiple imputation by chained equations with logistic-regression imputation (50 datasets), using age, sex, hypertension, treatment modality, trajectory class, baseline albumin, and discharge outcome as predictors; estimates were combined using Rubin’s rules. Patients included in versus excluded from the complete-case Model 3 were compared on available characteristics. Because several in-hospital complications could influence both albumin and neurological status, an exploratory model additionally adjusted for pneumonia, cerebral infarction, hydrocephalus, and intracranial infection; these post-baseline variables were not included in the primary model because they may also lie on the causal pathway. Classification uncertainty was examined using 500 pseudo-class datasets generated by sampling latent-class membership from each patient’s posterior probabilities and pooling the adjusted regression estimates. Finally, apparent discrimination and overall accuracy of the baseline clinical model and the clinical model plus trajectory class were summarized by the C-statistic and Brier score; 1,000 bootstrap resamples were used for optimism correction and calibration-slope estimation, with latent-class assignments held fixed during this model-validation step.

Additional sensitivity analyses addressed treatment-pathway confounding and exposure-outcome window coupling. Trajectory-outcome models were stratified by microsurgical clipping and endovascular treatment; the 9 patients who received both modalities were omitted from the stratum-specific analyses. Within each treatment stratum, models were adjusted for age, sex, hypertension, and baseline severity. A trajectory-by-treatment interaction was tested in the combined clipping/endovascular cohort using a two-degree-of-freedom likelihood-ratio test, and a direct adjusted comparison between the high-declining and low-persistent trajectories was estimated. To reduce overlap between albumin measurements collected through day 14 and discharge outcome ascertainment, the principal adjusted analysis was repeated among patients whose hospital stay exceeded 14 days. The full mRS 0–6 distribution across trajectory groups was compared using Pearson’s chi-square test.

## 3 Results

### 3.1 Study population and baseline characteristics

The patient selection process is shown in Figure 2. Of the 490 patients initially screened, 370 met the strict definition of index aSAH. After excluding 4 patients without serum albumin measurements within 48 h of admission and 48 patients with albumin measurements on fewer than three different days during the first 14 days, 318 patients were included in the final longitudinal trajectory analysis.

**Figure 2:**
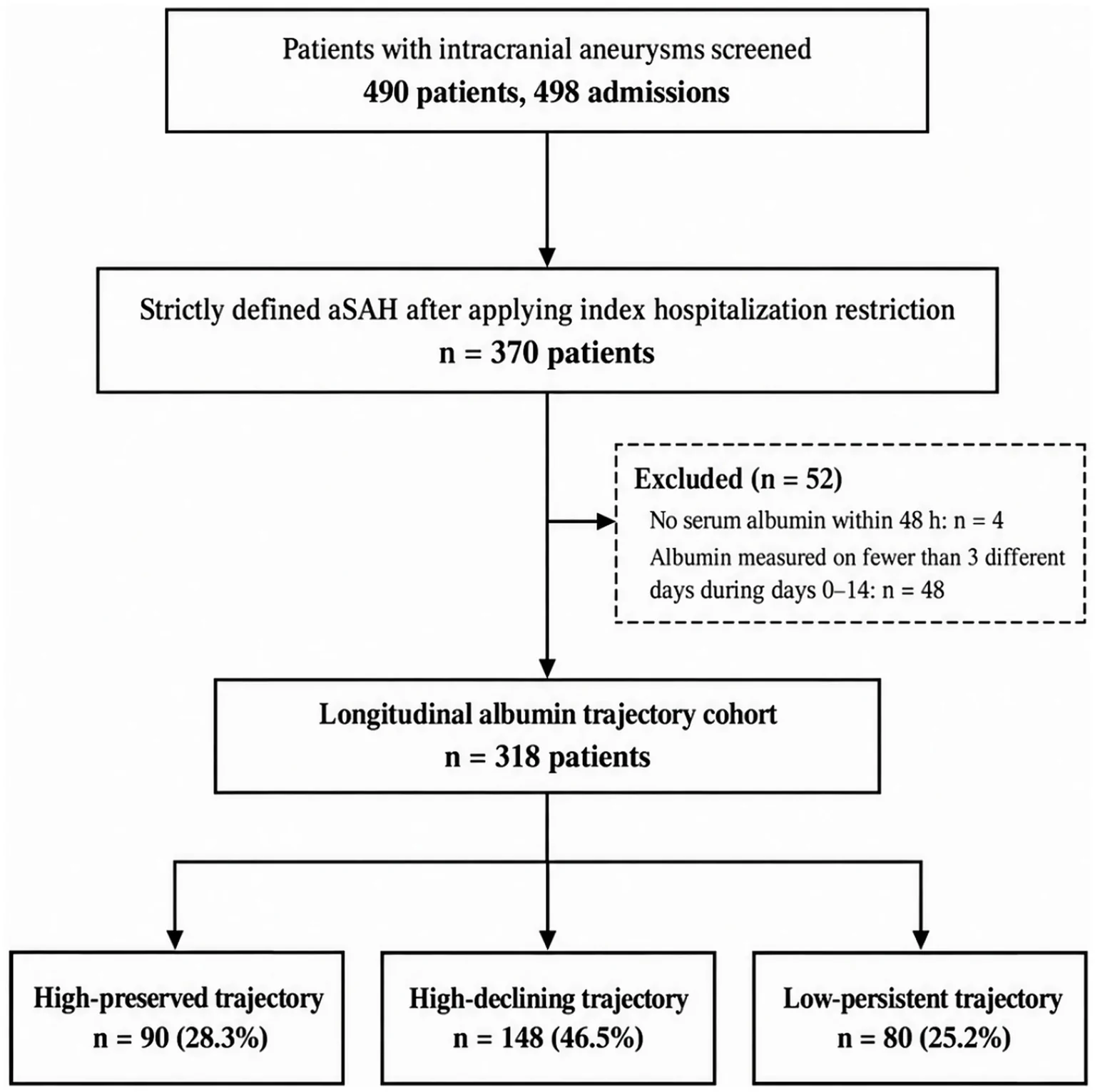
Study flow diagram for selection of the longitudinal albumin trajectory cohort.

The mean age of the study population was 60.5 ± 9.4 years, 112 patients (35.2%) were men, and 207 (65.1%) had hypertension. The median admission Glasgow Coma Scale (GCS) score was 14.0 (IQR, 11.0–14.0), and the median Hunt–Hess and modified Fisher grades were both 3.0. Baseline severity could be classified in 258 patients (81.1%): 185 were classified directly from an available Hunt–Hess grade and 73 used GCS as the fallback because Hunt–Hess was unavailable; 60 patients lacked both measures required for the composite indicator. Among the 258 classifiable patients, 137 (53.1%) met the poor-grade criterion. Microsurgical clipping was performed in 183 patients (57.5%), endovascular treatment in 126 (39.6%), and combined treatment in 9 (2.8%).

Baseline characteristics according to serum albumin trajectory group are summarized in Table 1. Patients in the high-preserved group were younger than those in the high-declining and low-persistent groups (56.9 ± 9.6 vs. 61.5 ± 9.2 vs. 62.5 ± 8.5 years, respectively; *p* < 0.001). As expected, baseline albumin concentrations also differed substantially among the groups (45.5 ± 3.4, 44.4 ± 3.3, and 36.7 ± 3.1 g/L, respectively; *p* < 0.001). Hunt–Hess grade differed among the trajectory groups (*p* = 0.006), as did the proportion of patients with poor-grade aSAH (39.4%, 56.2%, and 60.6%, respectively; *p* = 0.030). Treatment modality also varied significantly across the three groups (*p* < 0.001). In contrast, admission GCS score, modified Fisher grade, sex, and hypertension did not differ significantly among the trajectory groups.

**Table 1:** Baseline characteristics according to the selected serum albumin trajectory groups.

Variable	Overall	High-preserved	High-declining	Low-persistent	*p*-value
Age, years	60.5 ± 9.4	56.9 ± 9.6	61.5 ± 9.2	62.5 ± 8.5	<0.001
Baseline albumin within 48 h, g/L	42.7 ± 4.9	45.5 ± 3.4	44.4 ± 3.3	36.7 ± 3.1	<0.001
Admission GCS	14.0 (11.0–14.0)	14.0 (14.0–14.0)	14.0 (11.2–14.0)	14.0 (8.0–14.0)	0.089
Hunt–Hess grade	3.0 (2.0–3.0)	2.0 (2.0–3.0)	3.0 (2.0–3.0)	3.0 (2.0–4.0)	0.006
Modified Fisher grade	3.0 (2.0–3.0)	2.0 (2.0–3.0)	3.0 (2.0–3.0)	3.0 (2.0–4.0)	0.106
Male sex	112/318 (35.2%)	30/90 (33.3%)	48/148 (32.4%)	34/80 (42.5%)	0.286
Hypertension	207/318 (65.1%)	53/90 (58.9%)	102/148 (68.9%)	52/80 (65.0%)	0.290
Baseline severity indicator	137/258 (53.1%)	26/66 (39.4%)	68/121 (56.2%)	43/71 (60.6%)	0.030
Microsurgical clipping	183/318 (57.5%)	31/90 (34.4%)	107/148 (72.3%)	45/80 (56.2%)	<0.001
Endovascular treatment	126/318 (39.6%)	55/90 (61.1%)	38/148 (25.7%)	33/80 (41.2%)	
Both/combined treatment	9/318 (2.8%)	4/90 (4.4%)	3/148 (2.0%)	2/80 (2.5%)	

Data are presented as mean ± SD, median (IQR), or *n*/*N* (%). The baseline severity indicator was derived from Hunt–Hess grade when available (*n* = 185; poor grade defined as grade ≥3); when Hunt–Hess was unavailable, admission GCS was used as a fallback (*n* = 73; poor grade defined as GCS ≤12). Hunt–Hess and GCS were not treated as interchangeable measures. Sixty patients lacked both measures.

### 3.2 Identification of longitudinal serum albumin trajectories

The fit and classification statistics for the candidate latent class mixed models are presented in Table 2. The two-class natural cubic spline model with four degrees of freedom had the lowest BIC (9208.4), whereas the corresponding three-class model had a BIC of 9210.2 (ΔBIC = 1.72), indicating little separation between the two solutions on BIC alone. The three-class solution improved entropy from 0.306 to 0.520, although this still represents only moderate class separation, and all three classes contained at least 25% of the cohort. Class-specific average posterior probabilities were 0.798 for high-preserved, 0.772 for high-declining, and 0.771 for low-persistent. The proportions with assigned posterior probability ≥0.70 were 73.3%, 66.2%, and 62.5%, respectively, and the corresponding odds of correct classification were 10.01, 3.89, and 10.04. Although the four-class model yielded a higher entropy of 0.587, one class contained only 8 patients (2.5%). On balance, the three-class solution was retained for the main descriptive analysis, with classification uncertainty explicitly addressed in sensitivity analyses.

**Table 2:** Comparison of candidate latent class mixed models for longitudinal serum albumin.

Time function	Classes	Log likelihood	AIC	BIC	ΔBIC	Entropy	Class sizes	Average posterior probability	Selected
Cubic	2	−4593.3	9208.6	9249.9	41.5	0.655	55/263	0.770/0.927	
Linear	2	−4880.5	9775.1	9801.4	593.0	0.573	57/261	0.757/0.895	
Natural cubic spline (df = 3)	2	−4688.4	9394.8	9428.7	220.2	0.556	70/248	0.794/0.886	
Natural cubic spline (df = 4)	2	−4572.5	9167.1	9208.4	0.0	0.306	144/174	0.748/0.775	
Natural cubic spline (df = 4)	3	−4559.0	9150.0	9210.2	1.7	0.520	148/90 / 80	0.772/0.798/0.771	Yes
Natural cubic spline (df = 4)	4	−4548.2	9138.5	9217.5	9.0	0.587	91/113 / 106/8	0.779/0.766/0.763/0.808	
Quadratic	2	−4707.1	9432.2	9466.0	257.6	0.641	52/266	0.781/0.916	

All candidate models included a random intercept. The two-class natural cubic spline model had the lowest BIC; the selected three-class solution had ΔBIC = 1.7, indicating similar fit on BIC alone, but was retained because it separated clinically distinct patterns without creating a very small class. For the selected classes, average posterior probabilities were 0.798 (high-preserved), 0.772 (high-declining), and 0.771 (low-persistent); median assigned posterior probabilities were 0.839, 0.811, and 0.792; proportions with posterior probability ≥0.70 were 73.3%, 66.2%, and 62.5%; and odds of correct classification were 10.01, 3.89, and 10.04, respectively. Overall entropy was 0.520, indicating moderate separation.

The class-specific longitudinal serum albumin profiles with 95% confidence intervals are shown in Figure 1, together with the number of patients contributing a measurement on each study day. The high-preserved group included 90 patients (28.3%) and showed the highest initial albumin level followed by an early decline and relative stabilization. The high-declining group comprised 148 patients (46.5%) and showed a steeper early decrease to approximately 31–32 g/L, whereas the low-persistent group included 80 patients (25.2%) and began at a lower level that remained low. Measurement density declined over time; on day 14, albumin values were available for 9 high-preserved, 25 high-declining, and 11 low-persistent patients. Confidence intervals therefore widened toward later time points, particularly in the smaller groups.

### 3.3 Albumin trajectories and unfavorable neurological outcome

Overall, 114 of the 318 patients (35.8%) had an unfavorable neurological outcome at discharge, defined as a modified Rankin Scale (mRS) score ≥3. The distribution of discharge mRS scores across the three albumin trajectory groups is shown in Figure 3. Unfavorable outcomes occurred in 13 of 90 patients (14.4%) in the high-preserved group, compared with 67 of 148 (45.3%) in the high-declining group and 34 of 80 (42.5%) in the low-persistent group. The complete mRS 0–6 distributions were 12/42/23/6/4/2/1 in the high-preserved group, 17/40/24/18/26/23/0 in the high-declining group, and 10/25/11/5/15/14/0 in the low-persistent group. The distributions differed across trajectory groups (Pearson χ^2^ = 34.86, df = 12, *p* < 0.001; Figure 3).

**Figure 3:**
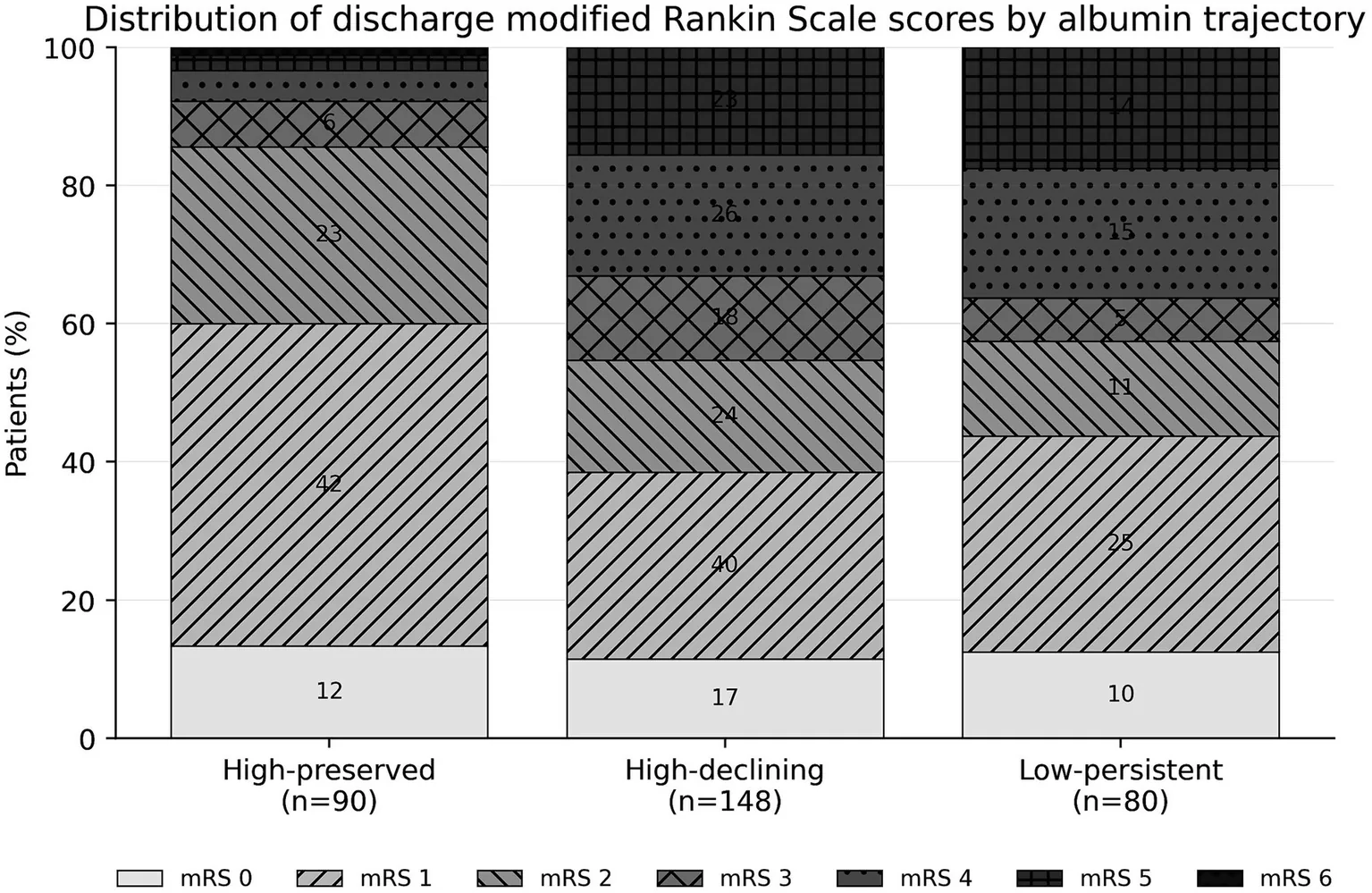
Distribution of discharge modified Rankin Scale (mRS) scores across the three serum albumin trajectory groups. The revised figure presents the full mRS 0–6 distribution as 100% stacked bars, with segment labels showing patient counts. Group distributions were compared using Pearson’s chi-square test (*χ*^2^ = 34.86, df = 12, *p* < 0.001).

The associations between serum albumin trajectory membership and unfavorable neurological outcome are summarized in Table 3. In the unadjusted model, compared with the high-preserved trajectory, the odds ratio (OR) was 4.90 (95% CI, 2.50–9.58; *p* < 0.001) for the high-declining group and 4.38 (95% CI, 2.10–9.14; *p* < 0.001) for the low-persistent group, with a significant overall association for trajectory class (global *p* < 0.001).

**Table 3:** Association between selected serum albumin trajectory group and unfavorable neurological outcome.

Model	Comparison	*n*	Events	OR (95% CI)	*P*-value	Global *P*	Adjustment
Unadjusted	High-declining vs. High-preserved	318	114	4.90 (2.50–9.58)	<0.001	<0.001	Trajectory group only
Unadjusted	Low-persistent vs. High-preserved	318	114	4.38 (2.10–9.14)	<0.001		
Model 1	High-declining vs. High-preserved	318	114	4.34 (2.11–8.91)	<0.001	<0.001	Age, sex, treatment modality
Model 1	Low-persistent vs. High-preserved	318	114	3.72 (1.73–8.02)	<0.001		
Model 2	High-declining vs. High-preserved	318	114	4.25 (2.06–8.77)	<0.001	<0.001	Model 1 + hypertension
Model 2	Low-persistent vs. High-preserved	318	114	3.73 (1.73–8.06)	<0.001		
Model 3	High-declining vs. High-preserved	258	96	3.65 (1.57–8.47)	0.003	0.007	Model 2 + baseline severity indicator
Model 3	Low-persistent vs. High-preserved	258	96	2.48 (1.02–6.02)	0.044		

Outcome: unfavorable neurological status at discharge, defined as mRS ≥3. The high-preserved trajectory was the reference group. Model 3 was the principal adjusted complete-case model; the baseline severity indicator primarily used Hunt–Hess grade and used GCS only when Hunt–Hess was unavailable. In Model 3, the direct adjusted comparison of high-declining versus low-persistent was OR 1.47 (95% CI, 0.76–2.83; *p* = 0.248). Sensitivity analyses are presented in Table 5.

The associations remained evident after adjustment for age, sex, and treatment modality. In Model 1, the adjusted ORs were 4.34 (95% CI, 2.11–8.91; *p* < 0.001) for the high-declining group and 3.72 (95% CI, 1.73–8.02; *p* = 0.001) for the low-persistent group. Further adjustment for hypertension produced little change in the estimates.

In the principal complete-case Model 3, which additionally accounted for the pragmatic baseline severity indicator, 258 patients with 96 unfavorable outcomes were included. Assigned trajectory class remained associated with neurological outcome (global *p* = 0.007). Compared with the high-preserved trajectory, the adjusted OR was 3.65 (95% CI, 1.57–8.47; *p* = 0.003) for the high-declining trajectory and 2.48 (95% CI, 1.02–6.02; *p* = 0.044) for the low-persistent trajectory. Because trajectory assignment used albumin values collected after admission, these estimates are interpreted as adjusted associations rather than evidence of an independent causal or predictive effect. Because both trajectory odds ratios use the high-preserved group as the common reference, the larger point estimate for the high-declining group should not be interpreted as evidence that it carries a higher risk than the low-persistent group. A direct adjusted comparison of high-declining versus low-persistent yielded an OR of 1.47 (95% CI, 0.76–2.83; *p* = 0.248) (Figure 4).

**Figure 4:**
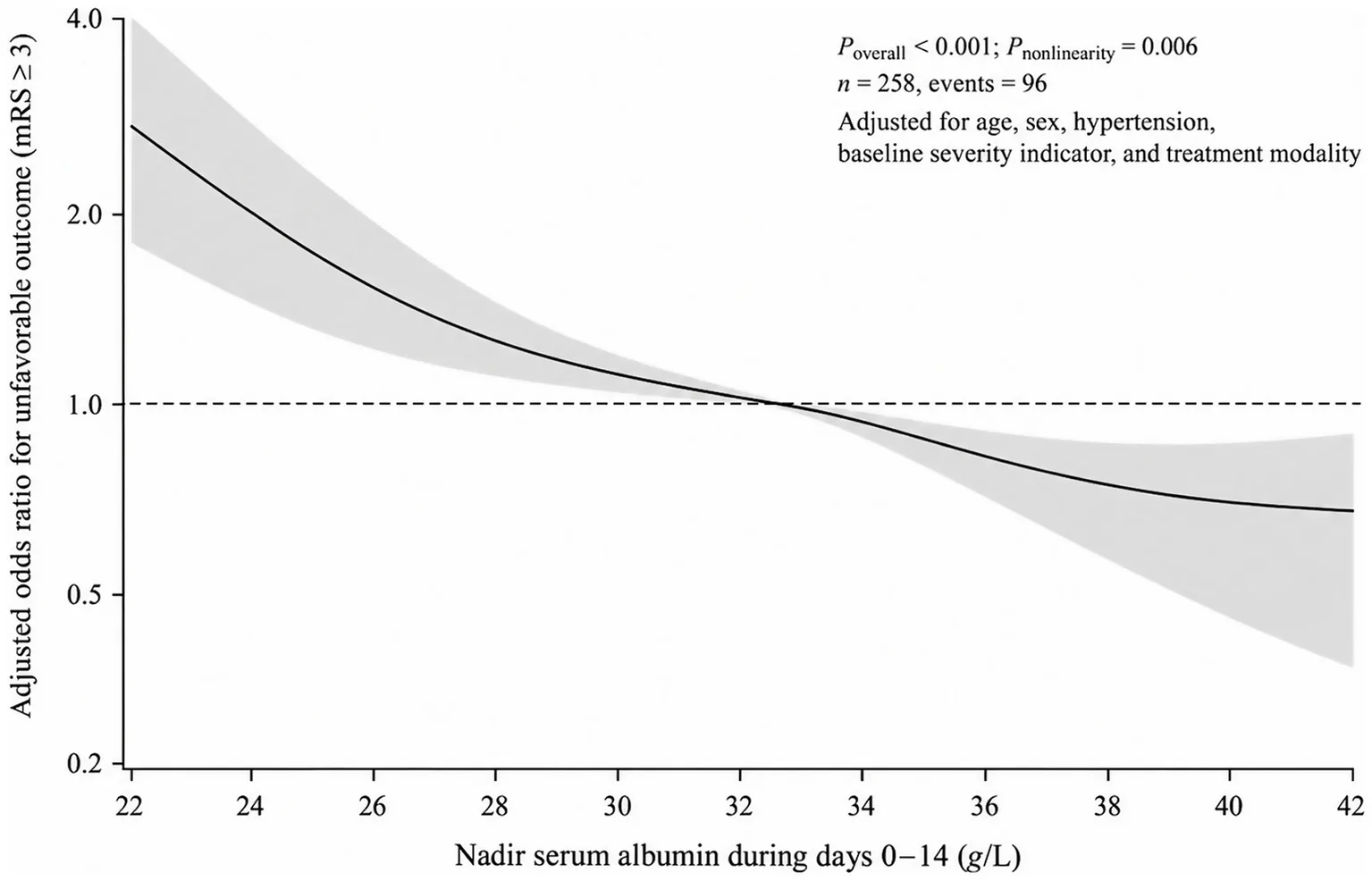
Restricted cubic spline association between nadir serum albumin during days 0–14 and unfavorable neurological outcome at discharge (mRS ≥3). The model was adjusted for age, sex, hypertension, the baseline severity indicator, and treatment modality. Because nadir albumin is post-baseline, the curve is interpreted as an adjusted association rather than a causal or validated predictive effect.

### 3.4 Association of baseline and nadir albumin with neurological outcome

The associations of baseline and nadir serum albumin with unfavorable neurological outcome are summarized in Table 4. Albumin measured within 48 h of admission was not significantly associated with unfavorable outcome after adjustment for age, sex, hypertension, baseline severity, and treatment modality. The adjusted OR per 1-g/L increase in baseline albumin was 0.97 (95% CI, 0.91–1.03; *p* = 0.286). The overall spline association was also not significant (*p* = 0.121), with no clear evidence of non-linearity (P for nonlinearity = 0.097). Nadir albumin during days 0–14 was strongly associated with unfavorable neurological status: the adjusted OR per 1-g/L increase was 0.76 (95% CI, 0.69–0.84; *p* < 0.001), and the restricted cubic spline showed an overall association (*p* < 0.001) with evidence of nonlinearity (*p* = 0.006). Because nadir albumin is a post-baseline measure that can be affected by the evolving clinical course and treatment, this result was treated as a time-varying association rather than a baseline prognostic effect.

**Table 4:** Restricted cubic spline analyses of serum albumin and unfavorable neurological outcome.

Albumin measure	*n*	Events	OR per 1 g/L increase (95% CI)	*P* for linear term	*P* overall (RCS)	*P* for nonlinearity
Baseline albumin within 48 h	258	96	0.97 (0.91–1.03)	0.286	0.121	0.097
Nadir albumin during days 0–14	258	96	0.76 (0.69–0.84)	<0.001	<0.001	0.006

Restricted cubic spline models were adjusted for age, sex, hypertension, the baseline severity indicator, and treatment modality. Nadir albumin was treated as a post-baseline time-varying marker associated with outcome, not as a baseline causal exposure or validated prognostic predictor.

### 3.5 Sensitivity analyses and robustness checks

The sensitivity analyses are summarized in Table 5. In the day-3 landmark analysis (*n* = 216; 84 events), each 1-g/L higher early nadir albumin was associated with lower odds of a subsequent unfavorable discharge outcome (adjusted OR, 0.86; 95% CI, 0.79–0.93; *p* < 0.001). The day-7 landmark analysis yielded a similar association (*n* = 250; 91 events; adjusted OR, 0.84; 95% CI, 0.77–0.91; *p* < 0.001). Restriction to the 185 patients with an available Hunt–Hess grade produced adjusted ORs of 4.37 (95% CI, 1.52–12.53; *p* = 0.006) for high-declining and 3.52 (95% CI, 1.17–10.61; *p* = 0.026) for low-persistent versus high-preserved (global *p* = 0.012). Multiple imputation of the missing baseline severity indicator using 50 datasets also produced associations in the same direction (high-declining OR, 4.34; 95% CI, 1.99–9.46; low-persistent OR, 3.45; 95% CI, 1.52–7.85; global *p* = 0.001).

**Table 5:** Sensitivity analyses addressing temporal sequence, treatment pathway, baseline severity, missingness, residual confounding, and latent-class uncertainty.

Analysis	*n*/events	Exposure or contrast	Adjusted OR (95% CI)	*P*-value	Global *P*
Day-3 landmark	216/84	Early nadir albumin, per 1 g/L higher	0.86 (0.79–0.93)	<0.001	–
Day-7 landmark	250/91	Early nadir albumin, per 1 g/L higher	0.84 (0.77–0.91)	<0.001	–
Hunt–Hess-only	185/61	High-declining vs. high-preserved	4.37 (1.52–12.53)	0.006	0.012
Hunt–Hess-only	185/61	Low-persistent vs. high-preserved	3.52 (1.17–10.61)	0.026	
Multiple imputation (*m* = 50)	318/114	High-declining vs. high-preserved	4.34 (1.99–9.46)	<0.001	0.001
Multiple imputation (*m* = 50)	318/114	Low-persistent vs. high-preserved	3.45 (1.52–7.85)	0.003	
+ available complications	258/96	High-declining vs. high-preserved	3.35 (1.37–8.15)	0.008	0.020
+ available complications	258/96	Low-persistent vs. high-preserved	2.09 (0.81–5.41)	0.129	
Posterior pseudo-class (500 draws)	258/96	High-declining vs. high-preserved	2.85 (1.04–7.79)	0.041	0.114
Posterior pseudo-class (500 draws)	258/96	Low-persistent vs. high-preserved	2.41 (0.87–6.71)	0.091	
Direct comparison (Model 3)	258/96	High-declining vs. low-persistent	1.47 (0.76–2.83)	0.248	–
Clipping stratum	150/64	High-declining vs. high-preserved	4.99 (1.25–19.84)	0.023	–
Clipping stratum	150/64	Low-persistent vs. high-preserved	4.27 (0.99–18.38)	0.051	–
Endovascular stratum	100/30	High-declining vs. high-preserved	5.75 (1.58–20.88)	0.008	–
Endovascular stratum	100/30	Low-persistent vs. high-preserved	1.77 (0.46–6.72)	0.405	–
Trajectory × treatment interaction	250/94	2-df likelihood-ratio test	—	0.453	–
Length of stay >14 days	198/74	High-declining vs. high-preserved	3.00 (1.12–8.07)	0.029	–
Length of stay >14 days	198/74	Low-persistent vs. high-preserved	1.98 (0.68–5.82)	0.212	–

Landmark models excluded patients discharged or dead on/before the landmark, required at least two albumin measurements within the window, and adjusted for age, sex, hypertension, treatment modality, and baseline severity. Treatment-stratified models adjusted for age, sex, hypertension, and baseline severity; the interaction test excluded the 9 patients who received both treatment modalities. The >14-day sensitivity analysis used the principal adjusted model among patients with length of stay >14 days. The complication model additionally included pneumonia, cerebral infarction, hydrocephalus, and intracranial infection; these variables may be mediators and were therefore used only for sensitivity analysis. Pseudo-class estimates were pooled across 500 posterior-probability draws.

Treatment-stratified analyses yielded similar direction for the high-declining trajectory. Among microsurgical clipping patients with classifiable baseline severity (*n* = 150; 64 events), the adjusted ORs versus high-preserved were 4.99 (95% CI, 1.25–19.84; *p* = 0.023) for high-declining and 4.27 (95% CI, 0.99–18.38; *p* = 0.051) for low-persistent. Among endovascularly treated patients (*n* = 100; 30 events), the corresponding ORs were 5.75 (95% CI, 1.58–20.88; *p* = 0.008) and 1.77 (95% CI, 0.46–6.72; *p* = 0.405). There was no statistical evidence of a trajectory-by-treatment interaction (*P* for interaction = 0.453), although confidence intervals were wide. In the sensitivity analysis restricted to patients hospitalized for more than 14 days (*n* = 198; 74 events), the adjusted OR was 3.00 (95% CI, 1.12–8.07; *p* = 0.029) for high-declining and 1.98 (95% CI, 0.68–5.82; *p* = 0.212) for low-persistent versus high-preserved.

In the exploratory model additionally adjusting for pneumonia, cerebral infarction, hydrocephalus, and intracranial infection, the high-declining estimate remained elevated (OR, 3.35; 95% CI, 1.37–8.15; *p* = 0.008), whereas the low-persistent estimate was attenuated (OR, 2.09; 95% CI, 0.81–5.41; *p* = 0.129; global *p* = 0.020). Propagating latent-class uncertainty through 500 posterior-probability pseudo-class draws further widened uncertainty: the pooled ORs were 2.85 (95% CI, 1.04–7.79; *p* = 0.041) for high-declining and 2.41 (95% CI, 0.87–6.71; *p* = 0.091) for low-persistent, with a global *p* = 0.114. These analyses indicate that the high-declining association was comparatively more robust, whereas inference for the low-persistent class was more sensitive to model specification and classification uncertainty.

### 3.6 Missing-data comparison and incremental model performance

Characteristics of the 258 patients included in Model 3 and the 60 excluded because baseline severity could not be classified are shown in Table 6. Age, sex, hypertension, treatment modality, nadir albumin, and the frequency of unfavorable outcome were similar between groups, whereas excluded patients had slightly higher baseline albumin and a different distribution of assigned trajectories. Multiple-imputation results were therefore used as a complementary analysis rather than assuming that complete-case missingness was negligible. Length of stay was also similar between groups (median 21.0 vs. 23.5 days; *p* = 0.229).

**Table 6:** Comparison of patients included in versus excluded from the principal complete-case Model 3.

Variable	Included (*n* = 258)	Excluded (*n* = 60)	*P*-value
Age, years	60.5 ± 9.7	60.3 ± 7.8	0.872
Male sex	89 (34.5%)	23 (38.3%)	0.681
Hypertension	163 (63.2%)	44 (73.3%)	0.182
Treatment: clipping/endovascular/both	150/100 / 8	33/26/1	0.707
Baseline albumin within 48 h, g/L	42.5 ± 4.9	43.9 ± 4.5	0.035
Nadir albumin during days 0–14, g/L	30.7 ± 4.0	31.6 ± 3.7	0.104
Trajectory: high-preserved/high-declining/low-persistent	66/121 / 71	24/27 / 9	0.036
Unfavorable outcome, mRS ≥ 3	96 (37.2%)	18 (30.0%)	0.368
Length of stay, days	21.0 (15.0–32.5)	23.5 (16.0–40.3)	0.229

Age and albumin measures were compared using Welch *t*-tests, length of stay using the Mann–Whitney *U* test, and categorical variables using chi-square tests. Exclusion from Model 3 was due to absence of both Hunt–Hess grade and admission GCS needed to construct the baseline severity indicator.

Model-performance comparisons are shown in Table 7. In the complete-case cohort, the clinical model containing age, sex, hypertension, treatment modality, and baseline severity had an apparent C-statistic of 0.754 and Brier score of 0.187. Addition of trajectory class increased the apparent C-statistic to 0.776 and reduced the Brier score to 0.180. After 1,000 bootstrap resamples with class assignments held fixed, optimism-corrected C-statistics were 0.735 and 0.753, respectively, and the corresponding calibration slopes were 0.87 and 0.85. Thus, trajectory class provided at most modest incremental model information in this dataset and should not be presented as a validated improvement in prognostic prediction.

**Table 7:** Incremental model performance in the complete-case cohort (*n* = 258).

Model	Apparent C-statistic	Apparent Brier score	Optimism-corrected C-statistic	Optimism-corrected Brier score	Bootstrap calibration slope
Clinical model	0.754	0.187	0.735	0.197	0.87
Clinical model + trajectory class	0.776	0.180	0.753	0.192	0.85

The clinical model included age, sex, hypertension, treatment modality, and the baseline severity indicator. Optimism correction and calibration slopes were estimated from 1,000 bootstrap resamples. Latent-class assignments were held fixed during bootstrap validation; this analysis therefore evaluates the downstream outcome model, not reproducibility of the LCMM class-discovery step.

## 4 Discussion

In this retrospective cohort, longitudinal modeling identified three serum albumin patterns during the first 14 days after aSAH. Assigned high-declining and low-persistent classes were associated with higher odds of discharge mRS ≥ 3 in the principal complete-case model, whereas albumin within 48 h was not associated with outcome after adjustment. The revised sensitivity analyses add important qualifications. Day-3 and day-7 landmark analyses showed that lower early-window nadir albumin preceded the subsequent discharge outcome, and Hunt–Hess-restricted and multiple-imputation analyses were broadly consistent with the main findings. However, adjustment for available in-hospital complications and explicit propagation of latent-class uncertainty attenuated some estimates, particularly for the low-persistent class. The results therefore support an association between evolving albumin patterns and short-term neurological status but not a causal or independently validated predictive interpretation.

The use of discharge mRS requires particular caution. Standardized 90-day mRS was not systematically available in the retrospective records, and discharge status can be influenced by length of stay, rehabilitation transfer, and local discharge practice. It may also create exposure-outcome window coupling because patients with more severe illness can remain hospitalized longer and contribute more albumin measurements. In the sensitivity analysis restricted to patients hospitalized for more than 14 days, so that discharge outcome ascertainment followed the full day 0–14 albumin window, the high-declining association remained evident while the low-persistent estimate was attenuated and imprecise (13). This analysis reduces, but does not eliminate, the timing concern and cannot substitute for a standardized 90-day outcome. Future prospective studies should use 90-day mRS as the principal functional endpoint.

Our results extend recent nutritional and inflammation-related research in aSAH. Nutritional risk assessed with mNUTRIC and composite scores such as HALP has been associated with functional outcome, complications, or mortality (5). Albumin itself has been linked to hospital-acquired infection, while D-dimer/albumin and albumin/fibrinogen ratios have been associated with disease severity and longer-term outcome (14). Other studies have shown that inflammatory markers measured before or after aneurysm treatment, including SII, SIRI, CRP/albumin ratio, peroxiredoxin 2, and neurofilament light chain, track with outcome or secondary complications (10). Collectively, this literature supports the view that systemic biological responses after aSAH may add prognostic information to established neurological grades (15).

The high-declining trajectory illustrates why a single admission measurement may be insufficient to describe the subsequent biochemical course. Patients in this group entered hospital with albumin values close to those of the high-preserved group but then showed a rapid fall toward approximately 31–32 g/L. Recent trajectory-based studies in aSAH have similarly shown that longitudinal glycemic patterns and time-varying physiological signals can identify clinically distinct courses that are obscured by isolated measurements. In the present study, the high-declining association also remained statistically evident in the complication-adjusted and posterior-probability sensitivity analyses, whereas estimates for the low-persistent class were more sensitive to uncertainty in class assignment. This distinction argues for interpreting the latent classes as probabilistic summaries of longitudinal patterns rather than fixed biological phenotypes (16).

Treatment pathway was an important potential confounder because clipping was substantially more frequent in the high-declining trajectory, whereas endovascular treatment was more common in the high-preserved trajectory. Treatment-stratified analyses showed an elevated association for the high-declining trajectory in both clipping and endovascular strata, and the formal interaction test was not significant. Thus, the observed association does not appear to be explained solely by treatment modality. Nevertheless, treatment selection was nonrandom, and residual confounding from procedural complexity, operative stress, blood loss, and perioperative management remains possible.

Albumin should not be interpreted solely as a marker of nutritional intake in acute neurological injury. During critical illness, inflammation-driven endothelial dysfunction and capillary leak can shift protein-rich fluid out of the intravascular compartment, while surgery, hemodilution, altered hepatic synthesis, increased catabolism, infection, and reduced intake may further lower circulating albumin. Contemporary reviews of capillary leak describe how systemic inflammation, glycocalyx injury, and endothelial hyperpermeability contribute to protein extravasation and edema formation (17). The observed trajectories in our cohort probably reflect a mixture of nutritional reserve, inflammatory burden, fluid balance, operative stress, and disease progression rather than a single biological mechanism.

Fluid and albumin management were part of routine clinical care rather than dictated by the study. The extracted retrospective dataset did not contain sufficiently complete structured information to verify a uniform institutional albumin-infusion policy across the entire study period or to quantify reliably the proportion of patients receiving exogenous albumin. Exogenous albumin administration and cumulative fluid balance therefore could not be incorporated as covariates, and treatment-related modification of measured albumin concentrations remains a potential source of residual confounding and trajectory misclassification.

There is also ongoing interest in whether albumin is therapeutically modifiable in SAH. A hemodynamically oriented protocol incorporating continuous albumin infusion was associated with lower delayed cerebral ischemia in one quasi-experimental study, and a randomized trial protocol published in 2025 is evaluating whether human albumin can improve clinical outcomes after aSAH (18, 19). These studies provide a rationale for continued investigation, but our observational data should not be interpreted as evidence that correcting hypoalbuminemia with albumin infusion will improve neurological recovery. A low or falling albumin concentration may primarily be a marker of systemic illness, and interventional efficacy requires randomized evidence (20).

The restricted cubic spline findings further emphasize the temporal distinction between admission albumin and values observed during the subsequent hospital course. Baseline albumin within 48 h was not significantly associated with unfavorable outcome after multivariable adjustment, whereas lower nadir albumin during days 0–14 was strongly associated with discharge status. Because nadir albumin can occur after infection, ischemic injury, surgery, fluid administration, nutritional intervention, or other clinical deterioration, reverse causation remains plausible. The day-3 and day-7 landmark analyses reduce this concern by restricting albumin exposure to an early window and excluding patients discharged or dead before the landmark; both analyses showed a consistent association between lower early nadir albumin and later discharge outcome. Nevertheless, a landmark analysis cannot exclude deterioration that occurred within the early window, so the findings remain associational.

From a clinical perspective, serum albumin has the advantage of being inexpensive and repeatedly available in routine care. The present results do not support using trajectory membership as a stand-alone treatment target, but they suggest that a rapid decline or persistently low albumin could prompt closer assessment of nutritional delivery, infection, inflammatory burden, fluid balance, organ function, and overall clinical deterioration (21). Such use would complement, rather than replace, established neurological grading and guideline-directed aSAH management.

The trajectory model itself warrants cautious interpretation. The two-class natural cubic spline model achieved the lowest BIC, while the selected three-class model was only 1.72 BIC points higher; this small difference provides little statistical evidence that one solution fits substantially better. The three-class solution was retained because it separated an initially preserved-but-declining pattern from a persistently low pattern, maintained all class sizes above 25%, and had class-specific average posterior probabilities of approximately 0.77–0.80. However, entropy was only 0.520, 33% of patients had an assigned posterior probability below 0.70, and the odds of correct classification for the high-declining class was lower than for the other two classes. Consistent with this uncertainty, posterior-probability pseudo-class analysis widened confidence intervals and rendered the overall class effect non-significant. The bootstrap analysis performed here validates only the downstream outcome model with class assignments held fixed; it does not establish reproducibility of the LCMM class-discovery step. Independent replication and, ideally, bootstrap refitting of the full LCMM are still required.

This study has several limitations. First, the retrospective, single-center design limits causal inference and generalizability. Second, trajectory membership and nadir albumin were defined using measurements collected through day 14, so values may have been influenced by complications or neurological deterioration that had already occurred. Landmark analyses and the >14-day hospitalization sensitivity analysis improved temporal ordering but could not eliminate reverse causation or exposure-outcome window coupling. Third, laboratory testing followed routine care rather than a fixed schedule; although Figure 1 reports 95% confidence intervals and daily measurement counts, later portions of the curves are supported by fewer observations and remain vulnerable to informative measurement frequency. Fourth, residual confounding is likely. We could additionally adjust for pneumonia, cerebral infarction, hydrocephalus, and intracranial infection, and treatment-stratified analyses did not show clear effect modification by clipping versus endovascular treatment; however, delayed cerebral ischemia as a separately adjudicated event, mechanical ventilation, ICU treatment intensity, cumulative fluid balance, nutritional delivery, hepatic/renal dysfunction, surgical complications, and exogenous albumin administration were not consistently available. Fifth, baseline severity was unavailable in 60 patients (18.9%). Included and excluded patients differed in baseline albumin and trajectory distribution; multiple imputation reduced reliance on complete-case analysis but cannot eliminate bias from unmeasured reasons for missing documentation. Sixth, latent-class separation was moderate and deterministic maximum-posterior assignment ignored uncertainty in the primary model; the pseudo-class analysis showed that this uncertainty materially affects precision. Seventh, some discharge mRS scores were retrospectively adjudicated. Although two investigators reviewed records independently and disagreements were resolved by consensus, their individual pre-consensus ratings were not retained, so weighted inter-rater kappa could not be reconstructed. Eighth, standardized 90-day mRS was not systematically available, and discharge mRS should be interpreted only as a short-term in-hospital endpoint. Finally, the modest apparent improvement in discrimination after adding trajectory class was attenuated after bootstrap correction, and the LCMM itself was not internally re-derived in bootstrap samples. Accordingly, the present results should be viewed as hypothesis-generating associations requiring prospective multicenter validation with standardized 90-day outcome assessment.

## 5 Conclusion

Three longitudinal serum albumin patterns were identified during the first 14 days after aSAH. Rapid decline from an initially preserved concentration and persistently low concentrations were associated with unfavorable neurological status at discharge in the primary analysis, but the magnitude and precision of these associations varied in sensitivity analyses that accounted for complications, treatment pathway, and latent-class uncertainty. Early-window landmark analyses showed that lower albumin within the first 3–7 days preceded worse discharge status, although reverse causation and residual confounding cannot be excluded. Serial albumin should therefore be considered a marker of the evolving systemic and clinical course rather than a stand-alone or independently validated prognostic predictor. These findings apply to short-term neurological status at hospital discharge and should not be extrapolated to standardized 90-day functional outcome without prospective follow-up validation.

## Data Availability

The datasets presented in this article are not readily available because the data from this study are available upon reasonable request from the corresponding authors. Requests to access the datasets should be directed to Youwen Gong, 1755144149@qq.com.
